# Climate Mismatch between Introduced Biological Control Agents and Their Invasive Host Plants: Improving Biological Control of Tropical Weeds in Temperate Regions

**DOI:** 10.3390/insects12060549

**Published:** 2021-06-12

**Authors:** Nathan E. Harms, Ian A. Knight, Paul D. Pratt, Angelica M. Reddy, Abhishek Mukherjee, Ping Gong, Julie Coetzee, S. Raghu, Rodrigo Diaz

**Affiliations:** 1 Aquatic Ecology and Invasive Species Branch, Environmental Laboratory, US Army Engineer Research and Development Center, Vicksburg, MS 39180, USA; Ian.A.Knight@usace.army.mil; 2 Invasive Species and Pollinator Health Research Unit, United States Department of Agriculture, Agricultural Research Service, Albany, CA 94710, USA; Paul.Pratt@usda.gov (P.D.P.); Angelica.Reddy@usda.gov (A.M.R.); 3 Indian Statistical Institute, Giridih, Jharkhand 815301, India; abhi.mukh@yahoo.com; 4 Environmental Processes Branch, Environmental Laboratory, US Army Engineer Research and Development Center, Vicksburg, MS 39180, USA; Ping.Gong@usace.army.mil; 5 Centre for Biological Control, Botany Department, Rhodes University, Grahamstown 6140, South Africa; Julie.Coetzee@ru.ac.za; 6 CSIRO Health & Biosecurity, Brisbane 4001, Australia; Raghu.Sathyamurthy@csiro.au; 7 Department of Entomology, Louisiana State University, Baton Rouge, LA 70803, USA; RDiaz@agcenter.lsu.edu

**Keywords:** biogeography, climate mismatches, enhanced traits, invasive plants, thermal physiology

## Abstract

**Simple Summary:**

Mismatched distributions between biological control agents and their host plants occur for a variety of reasons but are often linked to climate, specifically differences in their low-temperature tolerances. How to measure and use low-temperature tolerances of control agents to inform agent prioritization, selection for redistribution, or predict efficacy is vitally important, but has not been previously synthesized in a single source. We discuss causes of climate mismatches between agents and target weeds, the traditional and non-traditional approaches that could be used to decrease the degree of mismatch and improve control, and regulatory issues to consider when taking such approaches. We also discuss the variety of cold tolerance metrics, their measurement and ecological value, and the types of modeling that can be carried out to improve predictions about potential distributions of agents. We also briefly touch on molecular bases for cold tolerance and opportunities for improving cold tolerance of agents using modern molecular tools.

**Abstract:**

Many weed biological control programs suffer from large-scale spatial variation in success due to restricted distributions or abundances of agents in temperate climates. For some of the world’s worst aquatic weeds, agents are established but overwintering conditions limit their survival in higher latitudes or elevations. The resulting need is for new or improved site- or region-specific biological control tools. Here, we review this challenge with a focus on low-temperature limitations of agents and propose a roadmap for improving success. Investigations across spatial scales, from global (e.g., foreign exploration), to local (selective breeding), to individual organisms (molecular modification), are discussed. A combination of traditional (foreign) and non-traditional (introduced range) exploration may lead to the discovery and development of better-adapted agent genotypes. A multivariate approach using ecologically relevant metrics to quantify and compare cold tolerance among agent populations is likely required. These data can be used to inform environmental niche modeling combined with mechanistic modeling of species’ fundamental climate niches and life histories to predict where, when, and at what abundance agents will occur. Finally, synthetic and systems biology approaches in conjunction with advanced modern genomics, gene silencing and gene editing technologies may be used to identify and alter the expression of genes enhancing cold tolerance, but this technology in the context of weed biological control has not been fully explored.

## 1. Introduction

Management of invasive plants with biological control agents has the potential to be a cost-effective tool with long-lasting ecological and economic benefits. In many cases, the costs to develop and implement biological control are far outweighed by the persistent benefits that result from their programs [1,2,3,4]. To date, there have been over 1000 classical weed biological control programs worldwide, with high rates of success and limited non-target impacts [5,6,7]. Success and associated benefits of ongoing programs, however, can vary spatially and temporally and prompt additional research to identify the sources of variation.

**Figure 1:**
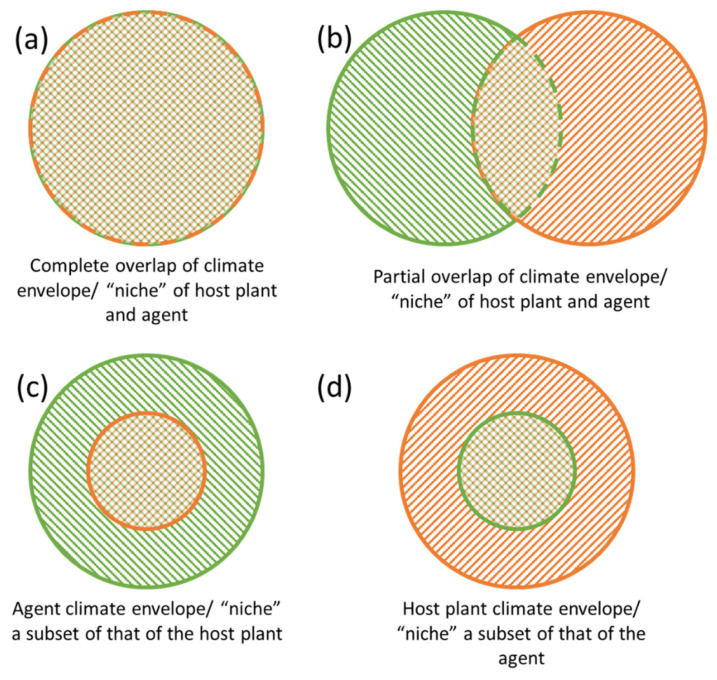
The four potential climate envelope overlap scenarios. In (**a**,**d**), there are minimal chances for climatic mismatches causing breakdown of biological control in the invaded range. In (**b**,**c**), opportunities for climatic mismatches in the invaded ranges result in only partial biological control across the invaded range. Green pattern = host plant; orange pattern = biological control agent.

The influence of geographic variation on the outcome of biological control programs is often related to the differential responses of agents and target weeds to biotic or abiotic factors, of which precipitation and/or temperature seem to be the most critical [8]. These differential responses may lead to cases where agent and host climate envelopes, and thus distributions, do not overlap completely (Figure 1b–d). Climate mismatches, scenarios when agents and hosts have different, not fully-overlapping climate envelopes generate patterns in which success may be inconsistent. There are numerous examples in the literature of climate mismatches fostering incomplete control in parts of the target weed’s introduced range (reviewed in [8]). Among the most problematic cases are aquatic weeds such as water hyacinth (*Pontederia* (= *Eichhornia*) *crassipes* (Mart.)), alligatorweed (*Alternanthera philoxeroides* (Mart.) Griseb.), and giant salvinia (*Salvinia molesta* D.S. Mitch.) that originate in the tropics but were introduced to, and readily invade, subtropical and temperate areas. Biological control programs for these invaders have resulted in regional success but agents are often more limited in their distribution than their target weeds [9,10,11,12] (i.e., Figure 1c). Efforts to improve biological control in underperforming regions has mostly been carried out by teams working without a cohesive conceptual framework that describes the problem, how to measure it, and possible strategies to address it.

Herein, we review climate mismatches in weed biological control programs with a specific focus on the world’s worst tropical and subtropical weeds. We provide background on causes of mismatch and examples with resulting control failures. We also discuss agent cold tolerance metrics and propose steps to improve biological control in cooler parts of the novel range into which they are released.

## 2. Why Does Climate Mismatch Occur?

Climate influences the abundance and distribution of organisms directly through mortality, altering developmental or reproductive success, or indirectly by acting on a food source or other habitat requirement [13]. When climatic mismatches occur between agents and hosts, it may be due to differences in physiological tolerances relative to climate extremes in the introduced range [8]. Specifically, temperature extremes and variability, rather than annual means, may be a better predictor of insect distributions and abundance because of the temporal scale at which important life history events typically occur (i.e., weeks to months, rather than years). Additionally, in areas near the range limits, organisms are presumably living near their physiological maxima [14]. Assuming a climatic analogue exists in the native range, the lack of agent cold tolerance may be due to sourcing agents from the wrong climatic region in the native range (e.g., [15]), climatic niche shifts [16], or reduced genetic diversity and/or loss of important cold-tolerant alleles during genetic bottlenecks, such as those encountered during laboratory rearing or field establishment [17].

### 2.1. Inadequate Exploration

Matching climates between the native and introduced ranges of a target weed is a foundational feature in biological control [18,19]. Attention was paid to climate compatibility when foreign exploration was conducted for agents of *A. philoxeroides*, the first widely successful aquatic weed biological control program [20,21]. However, because of regional variation in the magnitude of problems associated with *A. philoxeroides* infestations, early foreign exploration may have focused on the discovery of agents best adapted for Florida and other warm coastal states in the USA. The role of climate in the failure of biological control of *A. philoxeroides* in temperate areas was largely not addressed until the lack of control in higher latitudes was recognized [15,22] and the potential geographical distribution of the agent(s) was modeled [11]. The alligatorweed biological control system has since been the subject of continued interest [23,24].

Despite the modern capacity for environmental niche modelling (ENM; Section 5.1), climatic constraints of an agent are typically evaluated only after the regional failure of biological control is noted (e.g., [25,26]). Although there are recent examples of ENM to inform biological control exploration in the native range [27] or when transferring agents from one country to another [28], interest in using the potential (likely future) introduced range of a host to inform future biological control is increasing [29,30]. As a recent example, the potential geographic range of the invasive tropical soda apple (*Solanum viarum* Dunal) markedly exceeded that of its otherwise successful agent *Gratiana boliviana* Spaeth (Coleoptera: Chrysomelidae) (Figure 1c) [31]. Bet-hedging in this context, to mitigate possible future distributions of invaders may seem risky, particularly when developing agents is costly. Thus, responsible scientists must make decisions about how to allocate already scarce resources. At a minimum, understanding potential future distributions of target weeds in the introduced range can be used to direct opportunistic searches in different areas of the native range during surveys for other agents, and inform development of mitigation plans if invader distributions expand further (see Section 5).

### 2.2. Climate Niche Shifts and Expansions

Even if the appropriate native range is surveyed for natural enemies of target weeds, shifts or expansions in host or agent climate niches can constrain effective biological control. Niche shifts occur when the fundamental or realized niche of an organism changes, through release from biotic (e.g., natural enemies, competitors) or abiotic constraints (e.g., climate), or because of evolutionary processes [32,33,34]. In these cases, there may be no climate analogues in the native range that closely match the introduced range. If a host or agent is functionally excluded from a habitat in the native range, their introduced geographic range may be broader than their native one [35,36]. This issue must be acknowledged when using ENM to predict areas at risk of invasion [32,37]. Considerations of host plant niche shift and the implications for biological control in the literature are rare (but see [16]), but because niche expansion is postulated to be common among invasive species, it may have occurred in biological control systems and gone unrecognized. A likely example might be where a weed does not occupy all climatically suitable areas in the native range because of biogeographical barriers that prevent dispersal. A similar pattern could be generated by strong interactions with competitors [36] or shared natural enemies [38] that vary geographically and exclude the weed from some areas. For example, Kriticos and Brunel [39] found that the native potential distribution of *P. crassipes* under historical and future climate scenarios was smaller than predicted by environmental niche modelling. Because their modelling showed no apparent climatic reason why the species would be absent from suitable areas in its native range, they concluded that there must be some biotic or other non-climatic factor responsible for its absence there. Nevertheless, niche expansions of target weeds may pose a problem for sourcing agents. If the climatic range/tolerance of a weed is broader than its currently known native range (Figure 1c), foreign exploration cannot adequately cover all the climates of interest in the native range. Thus, agents located during surveys may not have suitable climate tolerances. This provides a strong case for assessing tolerances of agents alongside other evaluations (e.g., host range testing) to acquire a priori knowledge of an agent’s potential distribution before it is introduced.

### 2.3. Genetic Bottlenecks, Post-Introduction Evolution, and Hybrids

Founder effects, genetic bottlenecks, and loss of genetic diversity within target weed or agent populations may also account for differences in biological control success [17,40]. For plant populations, evolutionary processes that occur during or after introduction are important for invasion success and may give rise to unique genotypes, including hybrids that do not occur in the native range, or locally-adapted genotypes that perform well in a given environment [41,42,43]. The probability of genetic bottlenecks in biological control agent populations may be high, given that they are often originally sourced from one or a few sites [19], cultured for an extended period under relatively constant laboratory conditions, and survivors are used to inoculate field locations [44]. Agent laboratory cultures are periodically refreshed with new material to counteract the negative effects of small population size such as inbreeding and loss of alleles [45,46]. In New Zealand, genetic bottlenecks upon importation of the heather beetle (*Lochmaea suturalis* (Thomson) (Coleoptera: Chrysomelidae)) are thought to have led to poor establishment and overwintering survival in introduced populations [47]. In contrast, *Neochetina eichhorniae* Warner (Coleoptera: Brachyceridae) was imported into Australia from the USA where, subsequently, populations were found to be more cold tolerant [10] and less genetically diverse [48] than several USA populations. To reduce the importance of founder effects and reduced genetic diversity in introduced agent populations, multiple introductions from various source areas could be accomplished [48], although this may increase the regulatory burden for scientists if safety assessments are required for all unique source populations (see Section 7). Additionally, because different source areas may have unique host plant genotypes, an understanding of how agents will perform on genotypes present in the introduced range is important and can also be determined pre-release.

## 3. Addressing Climatic Mismatches

### 3.1. Traditional Approaches

The most common approach to address the limited distribution and abundance of existing agents involves returning to the foreign exploration phase. This may involve searching for new agent species that are better adapted to the habitat the weed occupies outside the realized distribution of the current agent(s). Surveying for new agent species, however, carries high levels of inherent uncertainties in the standard research phases: species discovery, identification, colonization in the laboratory, host range testing and impact, and establishment. An alternative approach includes sourcing new cold-hardy genotypes of an existing but regionally-limited agent.

Acquiring new genotypes may be a more effective and efficient use of limited resources as compared to investigating new agent species in the native range. Demand for expanding biological control across a target weed’s entire (or more temperate) range is often driven by the established agent’s observed efficacy in a subset of the range [10,49]. *Agasicles hygrophila* Selman and Vogt (Coleoptera: Chrysomelidae) provides complete control of *A. philoxeroides* in the plant’s most southern distribution in the eastern USA but provides inadequate control in more temperate zones [24,50]. Current research prioritizes acquiring new cold-hardy genotypes of *A. hygrophila* that are compatible with the weed’s temperate distribution over the development of new agents [51]. Attempts to expand the range of effective agents build on the axiom that the best predictor that an agent will successfully suppress the target weed is if the agent is effective elsewhere [52].

The search for cold-hardy genotypes may also be more efficient based on the assumption that intraspecific variation in temperature tolerance does not correlate with variation in host range (i.e., diet breadth). Two genotypes of the air potato beetle (*Lilioceris cheni* Gressit and Kimoto) (Coleoptera: Chrysomelidae), one collected in Nepal and another from China, exploit different environmental niches but share the same narrow host range [53,54]. Similarly, two genotypes of the planthopper (*Megamelus scutellaris* Berg) (Hemiptera: Delphacidae) introduced into the USA from two different climate sources, southern Argentina (cooler climate) and Paraguay (warmer climate), have the same narrow diet breadth [55,56]. Despite growing evidence host specificity is conserved across genotypes of a species (but see [57]), regulatory agencies commonly require experimental evidence of the new genotype’s host range prior to approving release. This requirement seems logical if host range testing of the original genotype lacked critical test plants present in the intended, more temperate range for the new genotype.

Searching for cold-tolerant genotypes is most amenable for target weeds and natural enemies that have large native distributions and when the original biotype was acquired from climatic zones that poorly represent the breadth of the weed’s invaded range. Climate modeling software, as described below (see Section 5), can aid in directing surveys to areas of the weed’s native range that are similar to the temperate region of interest. It remains less clear if natural enemy genotypes will possess cold tolerance alleles in the native range for weeds that have undergone niche shifts, considering the apparent lack of selection for temperate-tolerant genes in the plant’s native range. In these circumstances, it may be necessary to search for cold-tolerant genotypes along the plant’s most temperate extremes of the native range and assess compatibilities of life history parameters with the intended range.

### 3.2. Non-Traditional Approaches

An alternative to sourcing new genotypes is exploring the phenotypic plasticity of established agents [58]. Selective breeding of agricultural crops and livestock has been employed for millennia and is often cited as a tool to enhance biological control. Recent advances in the artificial selection of hygienic behavior in honey bees, wingless *Harmonia axyridis* (Pallas) (Coleoptera: Coccinellidae)*,* and drosophilid parasitoids underscores the promise of this approach for increasing biological control efficacy [58,59,60]. Similarly, genotype selection based on temperature tolerance may be a powerful tool to broaden environmental compatibility of weed biological control agents [61].

An initial step in this process includes evaluating the intraspecific variability in cold tolerances within the study population [60]. Cold-tolerant phenotypes can be selected through experimentation and mated with (1) other cold-tolerant phenotypes (strain selection), (2) individuals from the resident population (wild type) that lack cold tolerance (cross breeding), or (3) optimization for genetic variability for multiple heritable genes (e.g., both cold tolerance and fecundity). One commonly cited problem associated with artificial selection is the inadvertent loss of valued life history characteristics during breeding [62]. Therefore, after each generation following selective breeding for cold tolerance, the progeny should be retested using a suitable assay to determine heritability patterns for cold hardiness and other characteristics of interest.

Selective breeding for cold tolerance can be a lengthy process with risks of inbreeding and trait loss. Although a reduction in genetic diversity is still likely with a selective breeding program [63], the lengthy research and permitting phases of classical biological control can be bypassed when breeding is used to improve cold tolerance of established agents.

An alternative approach to selective breeding is the reliance on post-release evolution in introduced populations, assuming thermal tolerances are sufficient for initial establishment. After establishment, adaptation to cooler climates by natural selection may occur [64,65]. This is known to occur in both vertebrates [66] and invertebrates, including introduced biological control agents. The speed of natural selection in introduced populations will be influenced by the life history of the agent (i.e., univoltine or multivoltine) and the relative magnitude, and timing, of selection pressures. For multivoltine agents, selection for cold tolerance will occur only on the fall generation, and for long-term persistence of cold tolerance traits, will depend on whether there is a trade-off between cold tolerance and fitness during other times of the year. Microevolution can result in significant changes in thermal physiology traits over a relatively short period of time, especially for those species that are genetically diverse and have relatively short life cycles [67]. As such, rapid evolution of agents after release has been demonstrated [64]. For example, Coetzee, et al. [68] predicted that the mirid, *Eccritotarsus catarinensis* Carvalho (Hemiptera: Miridae), an agent of *P. crassipes*, would not establish in more temperate regions of South Africa following investigations of its thermal physiology. However, the agent did establish in areas with unfavorable climates [69]. This highlights that agents may become better adapted to local climates and more effective over time, provided sufficient genetic variation is present in traits likely to influence survival under different climatic conditions. Some traits are intrinsically more likely to be variable than others [70]. In order to sufficiently assess control success, it has been suggested that post-release evaluations should only be conducted 10 years after the first establishment of an agent, thus ensuring enough time has passed for the benefits of the agent to be realized [71]. However, to document trait evolution, it is necessary to monitor populations over time, for example before and after selective events (e.g., cold winters). A longer observation period may provide time for many agents to adapt to the thermal environment of the introduced range [69], and they may become more effective in those areas over time. Cold-adapted populations from the field could then be released to augment biological control at sites where agents have not yet established due to low-temperature tolerance limitations in those populations.

## 4. Cold Tolerance Metrics and Their Application

Insects are generally categorized with regard to low-temperature tolerance as freeze tolerant, freeze avoidant (or freeze intolerant), and chill susceptible [72,73,74]. Freeze-tolerant species endure freezing temperatures through the conversion of body water into extracellular ice and resistance to the interruption of vital processes (e.g., circulation and respiration), cell shrinkage, elevated osmolality, anoxia/ischemia, and other potential physical damages from ice [75]. Freeze-avoidant organisms overproduce antifreeze proteins and other cryoprotectants (e.g., sugars, polyols) to depress the temperature of spontaneous freezing (i.e., supercooling point) [76]. Chill-susceptible species are those lacking either of the preceding characteristics and probably encompass most tropical or subtropical insects introduced for weed biological control.

To measure and compare the properties underlying cold tolerance of insect species or genotypes, a number of metrics have been developed (Table 1). In general, metrics represent the measurement of failure (e.g., supercooling point, lower lethal temperatures) or performance (e.g., feeding rate, temperature-dependent development, fecundity). The level of effort required to measure metrics and their utility largely depends on the objectives of the program and life history adaptation of each insect.

### 4.1. Measures of Failure

Supercooling point (SCP) is the point at which intracellular water freezes and is measured by the release of a burst of heat, termed the latent heat of crystallization. Despite its common use to describe insect cold tolerance, SCP may actually be a poor measure for many systems [77] because some species experience significant temperature-related mortality at temperatures above their SCP [23,78,79,80]. SCP of tropical insects is rarely investigated, and few reports mention chill injury [49,80,81]. However, a small number of freeze-tolerant and freeze-avoidant species at tropical and subtropical latitudes have been documented [81]. Despite a lack of previous investigations, SCP may still provide a useful relative (i.e., comparative), rather than absolute, metric for addressing the issue of climate mismatches because climatic [82] or elevational gradients [83] in SCP have been found. Therefore, new surveys of native or introduced agent populations may lead to the discovery of variation in SCP. SCP is straightforward to measure and therefore a commonly used metric for determining relative differences in cold tolerance between populations/species [23,84]. Measuring it may therefore be worth including in attempts to define cold tolerance of agents, particularly if it is shown to correlate well with other, more relevant, metrics.

Lower lethal temperature (LLT) is a similar metric to SCP in that it provides a lower bound on the potential climatic range of a species. LLTs are frequently incorporated into environmental niche models to make distribution predictions for insects [78,79,85,86]. This metric is typically determined through experiments in which individuals are exposed to a range of low temperatures, generally above their SCP, for a predetermined length of time, after which the temperature is increased and individuals are allowed to recover. Sinclair et al. [72] described the LLT as the highest (cold) temperature which resulted in 100% mortality (LT_100_), though lethal temperatures may also be reported as LT_50_. Andersen et al. [80] identified both LLT and lethal time (LTi) as among the best predictors describing the distribution of tropical and temperate *Drosophila* species along a latitudinal gradient.

Beyond simple measurement of LLT, the interaction of exposure time and temperature must be acknowledged. The data from an integrative experimental approach, in which multiple exposure times and temperatures are crossed in a factorial design, can be used to create a three dimensional plot of survival over a range of temperature–time combinations. Using this approach, Nedvěd et al. [78] defined the sum of injurious temperatures (SIT), and the upper limit of chill injury zone (ULCIZ) as the degree-day relationship between exposure temperature and time which results in 50% mortality (SIT) and the lowest temperature which results in no chill injury, regardless of exposure duration (ULCIZ) [72,87]. This approach has not been widely used, likely due to the increased effort and logistical difficulties of fully crossing exposure time and temperature treatments; however, ULCIZ was recently used to model the potential geographic ranges of target weeds and agents in China [23,85].

Cold tolerance is also measured using non-lethal cold temperature response metrics. Among the most widely used are critical thermal minimum (CT_min_) and chill coma recovery time (CCRT). Chill coma is defined as the reversible loss of electrophysiological activity and movement [90]. Recovery time is the time until spontaneous movement or coordination is regained upon warming, generally after being at chill coma temperatures for a standard time (e.g., [49]). The temperature preceding loss of coordination is the CT_min_ (or CCIT), and is measured by slowly (<1 °C per minute) cooling an organism and recording the temperature when coordination is lost [72]. Though widely used as a metric of cold tolerance, the ecological significance of CT_min_ is rarely explicitly discussed in the literature. It is considered to mark the lower bound of activity, below which organisms do not perform any ecologically relevant behaviors (e.g., feeding, dispersal, reproduction, predator avoidance) [89,92,93], and for this reason has been used in predictive species distribution models [91]. Similar to SCP, CT_min_ can vary along latitudinal [80,94] and geographic gradients [92]. Therefore, research to determine agent interpopulation variability in CT_min_ may lead to the identification of better cold-adapted agent genotypes.

CCRT is often used as an alternative measurement to CT_min_ and for some species, the two may be correlated [93]. In the case the two are correlated, it may be argued that CCRT is less ecologically relevant but an adequate proxy to CT_min_ due to the logistical difficulties of the latter [79,90,95,96]. Regardless of its ecological relevance, CCRT does appear to provide a suitable indicator of chill tolerance. Anderson, et al. [97] observed that *Drosophila melanogaster* Meigen (Diptera: Drosophilidae) lines bred for shorter CCRT also had lower mortality following more severe cold shock treatments. Like CT_min_, CCRT can also vary among species from tropical and temperate origins [88], and along geographic gradients [94].

### 4.2. Measures of Performance

Cold tolerance is also investigated using metrics that may represent measures of performance, where response curves are generated over a range of non-lethal temperatures, and the list of focal traits (e.g., development time, metabolic rate, running/flight speed, fecundity, body size) varies. For example, among a suite of *N. eichhorniae* genotypes, Reddy et al. [10] found that fecundity at low temperatures was the only trait that varied between genotypes and would then be a valuable metric for prioritizing genotypes for release in temperate *P. crassipes* infestations.

Performance metrics may be particularly important for improving biological control. For example, in areas where agents successfully survive winter temperatures, impaired fecundity or long development times may prevent or delay populations from reaching levels needed for adequate control of the weed. Harms and Cronin [24] found that delayed spring activity of *A. hygrophila* due to cold winters resulted in reduced control of *A. philoxeroides*. Augustinus et al. [91] incorporated development time and egg hatching success into a predictive model to identify the potential distribution and performance of the agent *Ophraella communa* LeSage (Coleoptera: Chrysomelidae) on ragweed, *Ambrosia artemisiifolia* L. They largely validated their findings in field surveys and found high impacts to *A. artemisiifolia* in areas where optimal population growth of the agent was predicted to occur.

In screening and prioritizing cold-hardy genotypes, potential trade-offs may arise, particularly when couched in the context of global climate change and geographical shifting of climatically suitable areas. Although there is evidence that heat- or cold-hardening responses have trade-offs, these are often temporary and may reflect seasonal trends [92]. Lü, et al. [98] observed that heat and cold pre-treatment negatively affected longevity and survival for their respective crosses. In contrast, Anderson et al. [97] observed no changes in resistance to high-temperature exposure in *D. melanogaster* lines bred for reduced CCRT (see also [99]), and MacMillan, et al. [100] found that CCRT for *D. melanogaster* was not significantly correlated with tolerance to heat, desiccation, or starvation (but see [101]). Despite the lack of evidence for significant trade-offs between low and high-temperature tolerances, trade-offs may exist outside of heat tolerance. Shiota and Kimura [102] demonstrated slower walking speeds and pupal development of cold-tolerant drosophilids compared to their temperate and tropical congeners. Although trade-offs between high- and low-temperature tolerances are largely unsupported in the literature, trade-offs between thermal tolerance and performance deserve further study. If they are widespread, such trade-offs could have major implications for improving weed control in cool areas with cold-adapted agents.

## 5. Predicting Distributions of Agents and Hosts

To achieve successful management across the entire introduced range of a target weed, agents should ideally be widespread across the region and reach high population densities [103,104], either naturally or through augmentation (e.g., [105]). Therefore, understanding an agent’s potential distribution and its population dynamics is key for predicting biological control efficacy [89]. Such predictions could aid in recognizing climatic mismatches between target weeds and agents, identifying regions where suboptimal control should be expected, and guiding future research efforts to mitigate these issues.

### 5.1. Environmental Niche Modelling (ENM)

Biological control scientists increasingly rely on ENM to estimate potential distributions of target weeds and their potential agents across space and time [106,107]. Environmental niche modelling establishes a statistical relationship between known occurrences of a species and abiotic covariates (often climate variables) in the geographic region of interest, approximating the suitable climatic envelope for species’ presence. This relationship can be transferred spatially or temporally to estimate potential distribution in the corresponding area [108]. The application of ENM to predict the distributions of agents and their target weeds is inexpensive and can generate useful and testable predictions about the spatial extent of spread of potential agents [31]. Previous research has also suggested that the estimated probability of occurrence as generated by ENM may be useful as a proxy for population density ([109], but see [110] for an alternate view).

Environmental niche modelling can aid decision making at various stages of biological control development and provide crucial information for identifying and mitigating climate mismatch between weeds and agents. For example, ENM was used to examine the potential distribution of *G. boliviana* in the USA and it was found that because the distribution of *G. boliviana* would be restricted only to south Florida, further efforts to establish the insect above 29° N latitude would most likely fail [31]. Environmental niche modelling has also been used to prioritize agents according to their climatic suitability and potential for spread within the introduced range of a target weed. Sun et al. [111] constructed niche models simultaneously for ragweed (*A*. *artemisiifolia*) and its candidate agents to select one (or a combination of agents) with the widest coverage across the introduced range of the weed in Europe. They found that a large portion of the introduced range of *A. artemisiifolia* (central, northern and western Europe) would be unsuitable for any of the selected agents [111]. Although field validation is critical to assess this type of approach, pre-emptive application of niche models provides a basis for cost-effective evaluation of potential efficacy of agents before resource-intensive field experiments are conducted [111]. Sun et al. [112] used niche models to identify percent suitable areas in East Asia for *O. communa* and *Epiblema strenuana* (Walker) (Lepidoptera: Tortricidae), two North American agents of *A. artemisiifolia*. They found that *O. communa* would most likely have a larger overlap with the geographic range of *A. artemisiifolia* than *E. strenuana*, under both current and future climatic scenarios. Similarly, Minghetti et al. [113] used this approach to identify areas within the introduced range of *Solanum mauritianum* Scop. that were suitable for its agent, *Gargaphia decoris* Drake (Hemiptera: Tingidae). Their analysis revealed that the entire introduced range of *S. mauritianum* was suitable for *G. decoris* under both current and future climatic scenarios and therefore the prospect for successful control of *S. mauritianum* was promising. These ENM applications provide novel understanding of the potential climatic limitations in establishment of agents, allowing researchers to communicate more realistic expectations of success and to make projections that can be tested.

Environmental niche modelling can also be used to inform foreign exploration. For example, Russell et al. [49] used ENM to identify climatically similar regions in South America, the native range of *S. molesta*, to that of northern Louisiana, USA, to inform searches for cold-tolerant *C. salviniae* genotypes. Based on the climate suitability projection, they conducted exploration in areas not previously surveyed and were able to identify a *C. salviniae* genotype that is significantly more cold tolerant than the one previously released in the USA.

### 5.2. Mechanistic Modelling

Environmental niche modelling requires species occurrence records but if available records represent only a part of its full distribution, then models developed with such limited data will underestimate potential distributions [114]. To avoid challenges associated with implementing niche models using sparse occurrence data, numerous studies have advocated empirical estimation of a species’ tolerance to abiotic factors and to incorporate that information to predict agent distributions across a landscape [79].

The information generated from empirically-determined thermal requirements combined with temperature-driven models can be used to predict geographical limits of an agent’s distribution in the introduced range, and determine whether a climate mismatch with its target weed is likely to occur [115,116,117]. For example, degree-day models have been developed to predict distributions and the number of annual generations for a number of agents, including *Calophasia lunula* (Hufnagel) (Lepidoptera: Noctuidae) in Europe [118], *Hypocosmia pyrochroma* Jones (Lepidoptera: Pyralidae) in South Africa and Australia [119], and *Pareuchaetes insulata* Walker (Lepidoptera: Erebidae) in South Africa [120]. In a similar application, Manrique et al. [115] calculated the temperature-dependent developmental rate of *Episimus utilis* Zimmerman (Lepidoptera: Tortricidae) and then incorporated the degree-day requirement calculation into GIS software to map the number of generations the insect could complete across the weed’s introduced range in the USA. The model did not identify a climatic mismatch in Florida and predicted successful establishment of *E. utilis* throughout the state if released [115].

Because thermal requirement studies can be time consuming, they are often conducted post hoc to investigate establishment failure of agents [68]. In a departure from this trend, May and Coetzee [121] utilized a degree-day model to select appropriate climate-adapted agents for release in South Africa. They compared the thermal physiologies of two established agents of *P. crassipes*, *E*. *catarinensis* and *Niphograpta albiguttalis* Warren (Lepidoptera: Crambidae), with a candidate agent *M. scutellaris*. They determined *M. scutellaris* had a higher thermal requirement than *E. catarinensis* or *N. albiguttalis* and should therefore not be released in the more temperate Highveld regions of South Africa [121].

Critical thermal limits (see Section 4) and moisture stress of agents have been used to predict agent establishment across the introduced range of target weeds and to identify potential climatic mismatches. For example, Byrne et al. [122] determined the critical minimum temperature for development and the lower lethal humidity for egg hatching of *Gratiana spadicea* (Klug) (Coleoptera: Chrysomelidae) to investigate its failure to establish on *Solanum sisymbriifolium* Lam. in the Highveld region of South Africa. They found that stress generated by low humidity, not temperature, was the determining factor for establishment failure. Furthermore, Byrne et al. [79] identified a climate mismatch between the original collection location of *G. spadicea* in the weed’s native range in South America and the introduced Highveld region. Chidawanyika et al. [123] used MaxEnt [124] to generate projections of the current and future distributions of *Tradescantia fluminensis* Vell., and empirically-derived estimates of mean critical thermal limits of its agent *Neolema abbreviata* (Lacordaire) (Coleoptera: Chrysomelidae) to examine the likelihood of *N. abbreviata* to spread across the invaded range of the weed. Their predicted distribution confirmed that no climatic mismatch exists between the two and that *T. fluminensis* would likely spread further in the introduced range.

### 5.3. Fitted Process-Based Models

Apart from correlative and mechanistic models, hybrid or fitted process-based models (*sensu* Dormann, et al. [125]) incorporate both ecological knowledge and species-specific vital rates for improved prediction of an agent’s population density and ultimately its impact on the target weed [126]. This type of model avoids the uncertainty associated with a potential lack of correlation between species distribution models and population stability and density [109,110]. For example, the process-based mechanistic model, CLIMEX use species occurrence data to estimate specific parameters for model calibration [127,128]. Numerous studies have applied CLIMEX models to detect potential mismatches between the distributions of biocontrol agents and their target weeds, identify climatically suitable regions in the invaded range for release of biocontrol agents and prioritized habitats in the native range for future surveys [129,130,131]. By combining degree-day and CLIMEX models, Coetzee et al. [68] demonstrated that cold winters would hinder establishment of *E. catarinensis* in South Africa. Apart from insect biocontrol agents, CLIMEX model also was utilized to determine the potential worldwide spread of the rust fungus *Miyagia pseudosphaeria* (Mont.) Jørst., a biological control agent for *Sonchus* species [132]. The authors identified that cold climates would limit the spread of the fungus and releases in suboptimal climates would most likely fail.

In a novel two-step approach, Augustinus et al. [91] incorporated experimentally determined vital rates of a biocontrol agent with ENM for improving prediction of its population density and potential impact on the target weed. They conducted field experiments to determine the role of temperature and relative humidity on development time and egg hatching rate, respectively, of *O. communa*, a potential biocontrol agent of the common ragweed in Europe. The obtained relationship was combined with published potential distribution maps and factors influencing population growth were determined [90]. They concluded that mechanistic niche models can generate more accurate predictions of agent efficacy, and contribute to informed weed management. Although field validation is needed, they represent a promising tool to support agent selection, guide field release, and evaluation of their potential impacts for successful biocontrol of their target weeds.

## 6. Non-Classical Biological Control: Molecular Approaches to Addressing Climate Mismatch

Molecular tools have the potential to address climate mismatches by using them to identify cold tolerance mechanisms and then manipulate them to overcome thermal limitations of agents. In fact, the use of advanced biotechnology has received recent attention for its potentially significant contribution to invasive species management [133,134,135,136]. Modern molecular approaches such as synthetic and systems biology, in combination with advanced genomics, gene silencing, and genetic engineering, have been employed to identify, characterize, activate and engineer/transform specific cold resistance genes in plants and animals [137,138,139]. In the following sections, we briefly review the state-of-the-art of molecular mechanisms underlying cold tolerance and then we introduce two technical approaches with potential to enhance insect cold tolerance: gene silencing and gene editing.

### 6.1. Molecular Mechanisms Underlying Cold Tolerance

Cold tolerance has been studied for decades and its physiological and cellular consequences are well understood for a few taxa [140]. Insects have evolved two distinctive temporary physiological mechanisms for coping with detrimental effects of low temperature: seasonal (SCH) and rapid cold hardening (RCH) [141,142]. RCH is thermal acclimation that occurs within a time course of less than a day, in response to chilling below the developmental threshold [142], whereas SCH requires days or weeks of exposure for induction and governs the gradual, predictable transition to the overwintering phenotype [141]. 

A number of genes, proteins, and metabolites have been implicated as playing essential roles in cold acclimation and overwintering diapause. These include cryoprotectants (e.g., low-molecular-weight sugar alcohols, such as glycerol, sorbitol and inositol, and other classes of compounds such as trehalose and the amino acid proline) and transporters that are involved in cryoprotective mechanisms, heat-shock proteins and antioxidant components of conserved stress response pathways, non-coding microRNAs and protein kinase or phosphatase that are implicated in fast-acting, readily reversible and ATP-inexpensive regulation of cell cycle and hypometabolism [75,143]. Unfortunately, the underlying molecular mechanisms, especially the regulatory gene interaction networks responsible for cold stress resistance, are far from being understood at the genetic (i.e., allelic variations), transcription, translation, post-translational phosphorylation, and signal transduction levels [142]. In order to identify genes or proteins that respond to low-temperature exposure, a genomics approach integrating transcriptomics, proteomics and bioinformatics has resulted in the discovery of abundantly expressed heat-soluble proteins [144], aquaporins [145], damage suppressor (Dsup) [146], and taxon-specific intrinsically disordered proteins [147]. The information gleaned from such basic scientific studies could be used to study and enhance low-temperature tolerances in weed biological control agents.

### 6.2. Enhancing Cold Hardiness

Two popular and powerful molecular tools are often employed to identify genes responsible for a particular phenotype and then “program” desired traits in an organism of interest: gene silencing and genetic engineering (particularly genome editing and gene drive).

Gene silencing (also known as gene knockdown) is a general term describing the interruption or suppression of gene expression at transcriptional or post-transcriptional/translational levels. A wide variety of strategies have been applied to repress gene expression, ranging from hybridizing to target mRNA, to catalyzing the cleavage of target mRNA, and binding to target proteins. There are three well-characterized and naturally occurring RNA interference (RNAi) pathways, regulated by microRNA (miRNA), piwiRNA (piRNA) and small-interfering RNA (siRNA), respectively [148]. The siRNA-mediated gene expression inhibition pathway is the most explored for pest control applications. From an insect control perspective, vital genes whose suppression may lead to mortality are the preferred silencing targets [149]. In contrast, it would be desirable to enhance cold tolerance in weed biological control agents to induce siRNA-mediated activity disruption of genes that inhibit the synthesis of cryoprotectants or antifreeze proteins. This could be accomplished through microinjection or endogenous delivery, for example incorporated into diet pre-release, of double-stranded RNA (dsRNA) or artificial miRNA [150,151]. The primary technical hurdles to this approach are the identification of appropriate genetic targets (e.g., transcription factors that reversibly regulate cold-inducible genes) and scalability. Despite the paucity of reports on gene silencing-induced cold hardening in ectotherms, the prospect of this application is promising, given the maturity and variety of the gene silencing technologies [138,141]. One possible application scenario would be to treat the biological control agent with RNAi (e.g., through feeding of RNAi-containing diet or spraying an RNAi-containing body coat) to induce expression of genes conferring cold hardiness prior to field release late in the fall. Ideally, this trait (even though transiently induced, in comparison with permanent genetic alteration) would last until spring, allowing for early control of target weed populations the following year. This might be most useful in cases where agents are not typically available to release early in the year and would still require ongoing effort to maintain agent populations. However, given the transient nature of RNAi after administration, a better use might be for identifying mechanisms of cold tolerance by sequential knockdown of suspected important genes. Once genes and regulatory elements are identified, they could serve as targets for CRISPR/CAS9 directed genomic editing, ultimately resulting in changes at the genomic level which would be permanent and self-propagating.

Genetic engineering technologies have been routinely used to clone or modify existing genes, leading to the alteration of traits or phenotypes of interest. There are numerous reports on breeding cold tolerance in crop and horticultural plants for temperate locations [138,152]. The recent emergence and advancement of clustered regularly interspaced short palindromic repeat (CRISPR) technology [153] has revolutionized genome editing and genetic engineering [133,134,135]. The CRISPR system is based on an RNA-guided endonuclease discovered in bacteria [154], repurposed by researchers for high-fidelity genetic engineering [155]. CRISPR has been applied to improve multiple traits of fruit crops, including resistance to biotic and abiotic stresses, fruit quality improvement, and domestication [156]. CRISPR-based gene drives are capable of spreading trait-altering/conferring gene(s) into an entire population through sexual reproduction [138,140,141]. Thus, technical barriers to engineering genes conferring cold tolerance in agents appear low, and these approaches may contribute to reducing climate mismatches between introduced agents and their target weeds. To achieve this goal, further basic research is required to better understand molecular mechanisms underlying cold tolerance (see Section 6.1) and to sequence the genome of biocontrol agents and characterize genes relevant to cold acclimation, all of which are essential for identifying and choosing the target genes for editing. Although there exist mixed sentiments about releasing genetically modified insects into the environment [157,158], sentiment may ultimately depend on the perceived cost–benefit ratio of the introduction [159]. Thus, rather than provide a roadmap for releasing genetically modified biological control agents, we intend to highlight possibilities for identifying molecular bases of cold tolerance and the ways in which genetic modification could be used to enhance weed biological control given further investigation.

At present, the use of molecular tools to enhance classical biological control programs is still in its infancy. Critically, our understanding of cold hardening and cold tolerance responses in insects, in general, is still lacking. In order to take advantage of genetic engineering technologies, a better understanding of biochemical and regulatory mechanisms underlying cold tolerance is required. Thus, basic research on these mechanisms is needed to support future developments in this regard. Due to regulatory hurdles for field-releasing genetically modified organisms, it may be more practical to develop gene silencing technologies to transiently induce cold stress tolerance in agent species. The major challenges of an RNAi-based gene silencing approach include but are not limited to: identification of potential genetic targets, delivery methods of silencing molecules (triggers), off-target effects, and complexity of insect biology, which requires further in-depth mechanistic research and careful selection and testing of target genes [160]. Nevertheless, given the rapid advances in the understanding of molecular mechanisms for both SCH and RCH and such biotechnologies as genomics, gene silencing and genetic engineering, we remain optimistic about the future of applying molecular approaches to the development and enhancement of cold-hardy agents.

## 7. Regulatory Considerations

Classical biological control is a regulated enterprise worldwide, with many countries implementing formal permitting requirements at the agent export, import and release phases of the process [161]. Regulatory requirements are designed to improve security and safety but acquiring permits in a timely manner has been a significant obstacle that delays or in some cases entirely halts research efforts. However, the relevance of regulatory oversight is expected to be variable across the approaches to improve climatic compatibility described here. In the USA, the US Department of Agriculture (USDA) has national oversight for biological control research and requires that new foreign populations of agent species already introduced in the USA must be vetted in the same manner as novel agents. This indicates cold-adapted biotypes of an approved agent discovered during foreign surveys of new climate-matched areas would require data confirming their narrow host range and then subject to the same permitting process, as was the case with *M. scutellaris* collected from Paraguay for release against water hyacinth [55] (see Section 3.1). In South Africa, the Departments of Environment, Forestry and Fisheries, and Agriculture regulate the release of biological control agents following extensive host-specificity testing under quarantine conditions. Although there is no regulatory requirement in South Africa that new populations of an already approved agent undergo new testing, biological control practitioners there follow the International Code of Best Practices for Classical Biological Control of Weeds [162], which requires all populations of an agent collected from the native range to be tested in quarantine. For example, although the water hyacinth mirid, *E. catarinensis*, collected from Brazil, was approved for release in South Africa in 1996, another population collected from Peru in 1999 was subjected to limited host specificity testing and efficacy studies before it was released in 2007 [163].

In contrast, the redistribution of cold-hardy genotypes that have undergone selection for cold-tolerant alleles within the same country are expected to experience few regulatory hurdles. In the USA, redistribution between states requires USDA approval but this is a much reduced process and in some cases select agents are pre-approved [164]. In Australia, redistribution across all states is permit-free with the exception of Western Australia, where an additional interstate biosecurity permit is required [165]. In South Africa, approval for redistribution between provinces is not required [166]. It is also assumed that no permitting will be required for the release of artificially selected agents if source populations were collected in country. It remains unclear what permitting requirements will be needed for genetically modified agents, but there is precedent in the field use of genetically engineered plants, which are regulated in the USA by the USDA Biotechnology Regulatory Service (BRS) and in Australia by the Office of the Gene Technology Regulator (OGTR). It is clear that some research approaches for improving cold tolerance of agents are more time consuming than others. However, a shortened regulatory process may offset time constraints or funding limitations for select methods and thus should be considered when selecting an approach.

## 8. Conclusions and Recommendations

**Figure 2:**
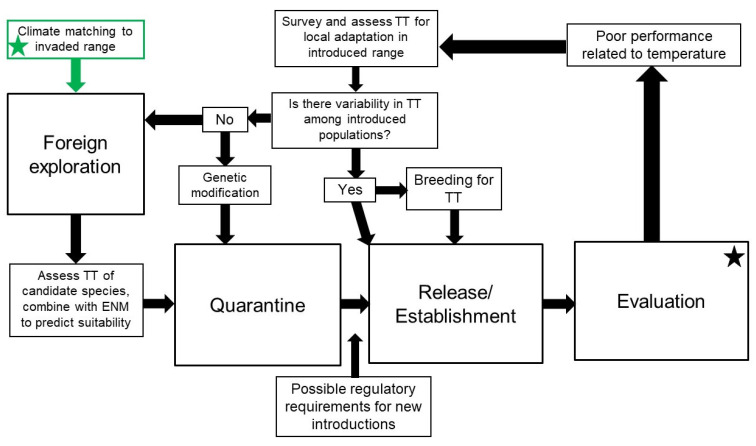
Proposed workflow for improving weed biological control outcomes where climate mismatches have been identified. Stars indicate workflow starting point for improving existing (black star) or developing new (green star) agents. Although not explicit in the diagram, at any point during agent development, it may be necessary to return to foreign exploration to source new agent genotypes or other candidate agents. TT = thermal tolerance; ENM = environmental niche modeling.

The sustainable, cost-savings benefits of biological control programs may be offset by the occurrence of climate mismatches between agents and target weeds in the introduced range. This can require a return to less ecologically and economically sustainable management techniques (e.g., herbicide treatment or mechanical removal of weeds) or additional research and associated resources to improve biological control success in these regions. Climate mismatches can occur because of inadequate exploration when sourcing potential agents, changes in host fundamental or realized climatic niche, or change in agent climatic niche during introduction due to reduced genetic diversity from either small initial population sizes or loss of alleles due to inbreeding. To confront and deal with the problem of climate mismatches, we propose a combination of monitoring, experimental, and exploratory approaches (Figure 2).

When developing new agents, we suggest climate matching-informed foreign exploration, ideally followed by low-effort but ecologically relevant assessments of thermal tolerances (Table 1) before or during the quarantine phase, combined with ENM of both agent and host to predict potential distribution patterns in the introduced range and identify potential for incomplete overlap. If the results of this exercise are positive (i.e., the agent is predicted to be abundant and provide control in high-priority areas), then agent development should proceed as normal. If control is likely to be limited geographically, and that limitation is unacceptable for goals of the control program, then either multiple climate-adapted genotypes or other candidate agents should be assessed.

If geographic variability in control is observed (and climate limitations are suspected as the cause) in an established program, then either traditional or non-traditional approaches can be undertaken to improve success. A return to the target weeds’ native range for additional exploration with climate matching in mind would potentially lead to sourcing agents that are pre-adapted to the environment in the introduced range and would then be developed following a similar workflow as new agents (see Section 3.1). In contrast, surveys of established agent populations throughout the introduced range, followed by screening for the desired traits (e.g., cold tolerance) may lead to agents that can be selectively mass reared and distributed into cooler regions (see Section 3.2). Regulatory actions required to redistribute agents will depend on where the agents are collected and where they are being moved, and what regulations there are for movement across political boundaries within a country (see Section 7). There may arise instances when better-adapted agents or biotypes cannot be identified in the wild type. In these cases, molecular tools might be employed to develop existing agents to perform in climates to which they are not adapted (see Section 6). Though significant work remains to be carried out regarding the physiology of traits such as cold tolerance, molecular tools such as CRISPR or RNAi to either introduce novel traits or altering mechanistic pathways associated with cold tolerance traits are promising in their outlook. Currently, these molecular approaches face significant social and regulatory hurdles, but are presented as a forward-looking solution if other approaches fail.

Our goal in this review is to clearly define the problem of climate mismatches for weed biological control agents, with focus on the use of tropical agent species in subtropical and temperate regions. Although there are myriad examples of how climate mismatches stymie biological control efforts, and in recent years the tools and concepts we discuss have been employed in finding solutions, no definite proposed framework for addressing these issues existed. We suggest the combination of traditional and non-traditional exploration, combined with empirical estimation of thermal tolerances to inform distribution modeling will help prevent future climate mismatches, address current ones, and contribute to the broader literature on species interactions, their distributions, and the importance of climate as a moderator of these interactions. We also suggest that the value of this framework be periodically reviewed and refined as its utility is tested in ongoing biological control programs.

## Figures and Tables

**Table 1:** Metrics used to describe cold tolerance of insects.

Cold Tolerance Metric	What It Measures	Effort Required	Ecological Relevance	Example Reference
**Super Cooling Point (SCP)**	Failure; the temperature at which ice crystals form in insect tissues.	Low	Delineates lower bound for freeze-intolerant taxa. Not useful for chill-susceptible taxa, as most will have died before supercooling. Not useful for freeze-tolerant taxa, as mortality will depend on temperatures and exposure times below the SCP. May be correlated with other cold tolerance metrics. Useful for distinguishing chill-susceptible and freeze-avoidant taxa, and quick comparison among populations.	Zachariassen [88]
**Lower Lethal Temperature (LLT)**	Failure; the highest (cold) temperature which results in a prescribed rate of mortality (e.g., LT_50_, LT_100_).	Low	Delineates lower bound for all cold tolerance categories, but is confounded by exposure time. Will be equal to the SCP for freeze-avoidant taxa.	Sinclair et al. [72]
**Lethal Time (LTi)**	Failure; the length of time at a given temperature which results in a prescribed rate of mortality.	Low	Confounded with LLT; useful for comparing relative cold tolerance among populations.	Andersen et al. [80]
**Sum of Injurious Temperatures (SIT)**	Failure; the degree-day relationship between exposure temperature and time which results in 50% mortality.	High	SIT and ULCIZ are determined together, and represent a comprehensive measure of chill induced mortality. ULCIZ delineates the isotherms between which there should be no expected cold-related mortality. Most relevant for delineating bounds of chill-susceptible taxa.	Nedvěd et al. [78]
**Upper Limit of Chill Injury Zone (ULCIZ)**	Failure; the lowest temperature which results in no chill injury, regardless of exposure duration.	High		Zhao et al. [23]
**Critical Thermal Minimum (CT_min_)/Chill Coma Induction Temperature (CCIT)**	Failure; the temperature, in a decreasing ramp, preceding loss of coordination (i.e., chill coma).	Medium	CCIT and CCRT are best used as relative measures of cold tolerance among populations. Due to their relative ease to measure, these may be useful for screening for cold tolerance traits; however, they are of limited use for species distribution models (SDM). CCRT may be preferable due to its relative ease to conduct.	Andersen et al. [89]
**Chill Coma Recovery Time (CCRT)**	Failure; the time until spontaneous movement or coordination is regained upon warming, after induction of chill coma.	Low		Gibert et al. [90]
**Temperature-Dependent Development (TDD)**	Performance; rate of development along a temperature gradient.	High	Development time determines how quickly agents can build to effective population sizes, both within a single generation and over multiple generations. Can be used to model performance within a SDM.	Augustinus et al. [91]
**Temperature-Dependent Performance (fecundity, etc.)**	Performance; feeding, fecundity along a temperature gradient.	High	Similar to TDD; can predict reproductive success as well as potential impact. Has also been used in SDMs.	Reddy et al. [10]

## Data Availability

Not applicable.
